# Paternalism in Historical Context: Helmet and Seatbelt Legislation in the UK

**DOI:** 10.1093/phe/phad001

**Published:** 2023-03-13

**Authors:** Janet Weston

**Affiliations:** London School of Hygiene and Tropical Medicine, London, UK

## Abstract

Paternalism is a frequent source of anxiety and scholarly enquiry within public health. This article examines debate in the UK from the 1950s to the early 1980s about two quintessentially paternalistic laws: those making it compulsory to use a motorcycle helmet, and a car seatbelt. This kind of historical analysis, looking at change over time and the circumstances that prevent or enable such change, draws attention to two significant features: the contingent nature of that which is perceived as paternalistic and therefore objectionable, and the wide range of arguments that can be marshalled for and against. It suggests that paternalism became a particularly disruptive accusation in the UK of the 1970s in relation to seatbelts, thanks to the population that would be affected and the wider socio-political context. It also suggests that arguments about the social cost of death and injury on the roads, along with overt acceptance that some element of paternalism could be acceptable, proved influential—as was the sense of inevitability that 10 years of regular debate helped to create.

Paternalism and its place in public health is contentious. Put simply, paternalism is commonly understood as an infringement of a person’s freedom or autonomy, for that person’s own good. To call something paternalistic is now usually a criticism, and one often levelled at public health interventions. In response, there have been many suggestions of ways to rebut or reconceptualise such a criticism. These have included calls to rethink our understandings of autonomy or self-determination, to refute the belief that paternalism is necessarily unacceptable, to destabilise some of the assumptions that underpin current debate, or to challenge the central role of paternalism within public health evaluations (Brännmark 2018; Carter *et al.*, 2015; Coggon 2020; Gostin and Gostin 2009; Holland 2009; Nys 2008; Wilson 2011).

Laws that penalise people who fail to wear a helmet when riding a motorcycle or a seatbelt in a car are often cited as quintessential examples of paternalism, whether in public health specifically or public policy more generally (Coons and Weber 2013; Coggon, Syrett, and Viens 2017; Flanigan 2017; Brännmark 2018). Such laws are not currently seen as controversial in the UK, although helmet laws have been fiercely contested and in some cases repealed in the USA (Jones and Bayer 2007). Helmet and seatbelt laws are usually said to be ethically acceptable because the infringement of individual freedom (to choose freely whether or not to use the helmet or seatbelt) is minimal, and the gains in terms of reduced chances of death or serious injury are substantial, creating a morally tolerable trade-off (Nuffield Council on Bioethics 2007).

To contribute to understandings of paternalism as a point of contention within public health, this article examines debate in the UK from the 1950s to the early 1980s about helmet and seatbelt laws. It focuses on parliamentary discussion rather than the more behind-the-scenes processes of policy development and delivery, which deserve separate attention. It uses Hansard, the official record of parliamentary debate in Westminster, supplemented by government archives and media coverage. This kind of historical analysis, looking at change (or lack thereof) over time and the circumstances that enable or prevent such change, directs attention towards two significant features of the public debate: the contingent nature of that which is perceived as paternalistic and therefore objectionable, and the range of arguments (with variable impact) that can be marshalled for and against seemingly paternalistic measures. It suggests that paternalism became a particularly disruptive accusation in the UK in the 1970s, when political instability coincided with rising popular individualism, reconfigurations of the role of the state, and greater emphasis on individual responsibility and education rather than compulsion. It also suggests that framing such laws as *both* (and sometimes simultaneously) ‘not paternalistic’ and ‘justifiably paternalistic’ was helpful in rendering them acceptable.

Historians of road safety have characterised the period from the end of the nineteenth century through to the dawn of the 1970s as one during which laws and regulations favoured private motorists, at the expense of pedestrians and other road-users (Luckin and Sheen 2009; Pooley 2021). Describing the opponents of road safety laws over these decades as ‘unreconstructed libertarians’ (Luckin 2010, 373), existing historical accounts gladly note their reduced number and influence by the 1960s, and attribute their decline to a combination of factors: technological innovation and mounting evidence surrounding collisions and safety; dramatically rising numbers of road users jostling for space; canny media management from safety advocates; and growing public pressure to deal with high rates of death and injury. Motorists, in this account, were finally compelled to yield by the late 1960s, and consensus over road safety could replace conflict.

The history of helmet and seatbelt laws and the debate over paternalism disrupts this picture, where conflict in the 1970s replaced prior consensus. A summary of successful and unsuccessful attempts to introduce helmet and seatbelts laws in the UK is given in Figures 1 and 2. Primary legislation empowering the transport secretary to make motorcycle helmets compulsory was passed in 1962, and this power was taken up in 1973: motorcyclists failing to wear helmets would incur a fine, and failure to pay could mean imprisonment. The same year saw the first public step towards a seatbelt law. Car manufacturers had been required to fit new vehicles with front seatbelts since 1965, in the hope that the constant presence of a belt would lead to habitual use, but even after intensive advertising only 30 per cent of drivers were doing so by the early 1970s.^1^ Following the announcement of a formal consultation in 1973,^2^ the question of compulsory use immediately became extremely controversial and was ultimately left to a free vote in Parliament the same way as abortion and the death penalty, framed as a matter of personal morality that transcended party politics. Between 1973 and 1981, under both Labour and Conservative governments, no fewer than ten attempts were made to pass a seatbelt law in the UK. Over these years, most of the UK’s European neighbours joined Australia in passing such a law. Failure to wear a belt finally became an offence in the UK 1983 thanks to the Transport Act 1981 and Motor Vehicles (Wearing of Seatbelts) Regulations 1982.

**Figure 1.**
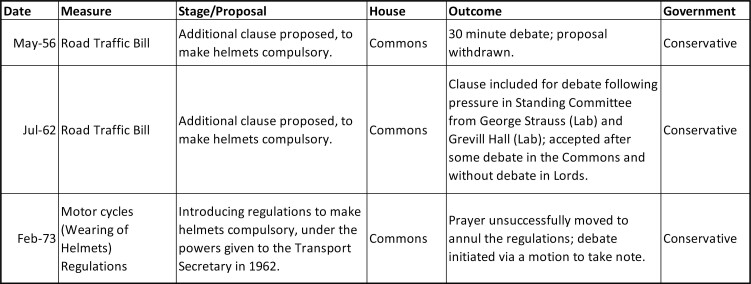
Successful and unsuccessful attempts to make motorcycle helmets compulsory.

**Figure 2.**
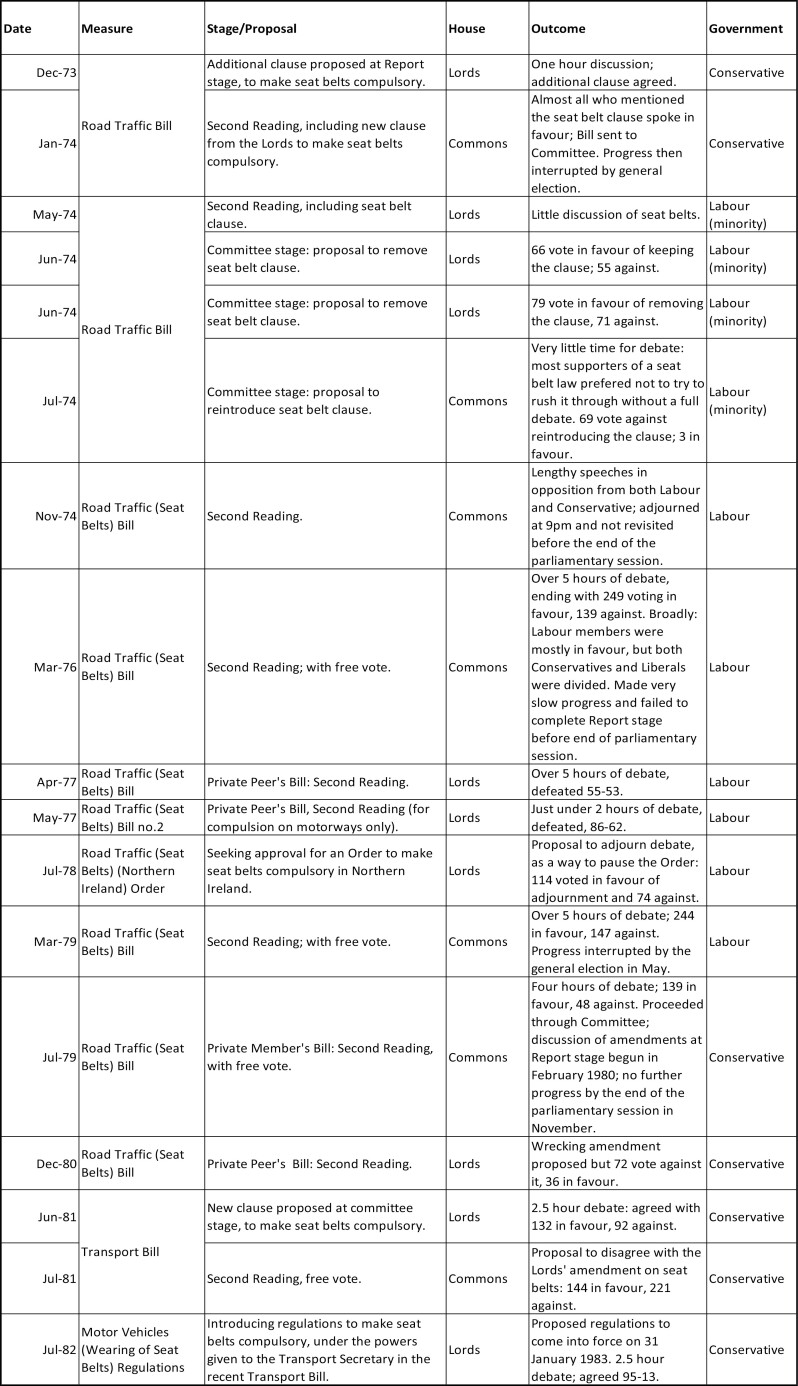
Successful and unsuccessful attempts to make car seatbelts compulsory.

Analysis in the 1980s attributed the arrival of a UK seatbelt law to the absence of industry opposition along with support from a range of professional groups (Leichter 1986). Little has been said, though, about why a seatbelt law–unlike a helmet law–became so morally loaded and generated such insistent opposition in the first place. I argue here that the contrasting trajectories of the two measures reflected not only the different constituencies that would be affected by them, but also the different socio-political context in which they were first proposed: by the 1970s, accusations of paternalism could be particularly damaging. This led to regular discussions about the rights and wrongs of a seatbelt law throughout the decade, during which two defences against the accusation of paternalism were raised: firstly, that the proposal was *not* paternalistic at all, and secondly, that it was, but was nonetheless justified. Both strands of argument proved influential.

## Helmets and Acceptable Paternalism

Motorcycle helmets had begun to prove their worth by the early 1940s (Cairns, 1941), and their design was developed and improved after the Second World War as the number of motorcyclists rose. The idea of a helmet law was first floated in the early 1950s and met with ambivalence. Amidst uncertainty about the quality of existing helmets, politicians felt that information and encouragement was more ‘in accord with the way we try to do things in this country’^3^–continuing a long tradition of characterising anything akin to policing health as ‘”foreign” to English political mores’, despite an equally long history of such policing (Carroll 2002, 491). Persistent concern about low-quality helmets and the number of motorcyclists killed and injured prompted in 1956 a first attempt to pass a law that would have provided for a minimum helmet standard as well as compulsory use. The short debate acknowledged the ‘really controversial matter,’ about paternalism: whether ‘a man has a right to kill himself’ by not wearing a helmet if he so chose, or whether the government had a responsibility to ‘protect the fool from his own folly.’^4^ Several speakers including the transport secretary were not convinced that compulsion was appropriate, but all could agree on the need for improving helmet standards and advocating for their use.

Six years later, with around 30 per cent of motorcyclists still not wearing helmets and over 1,500 fatalities and 26,000 serious injuries per annum, some were convinced that persuasion was not enough.^5^ Under pressure from a handful of Labour Members of Parliament (MPs), the Conservative transport secretary agreed to support the inclusion of permissive powers within his Road Traffic Bill of 1962, allowing for helmets to become compulsory in future. This received cross-party support, and only two MPs objected.^6^ Motoring associations muttered that it was ‘taking a sledge-hammer to crack a walnut,’ given the relatively high rates of helmet-wearing (*The Times* 1962), and some MPs reported receiving letters of protest from motorcyclists, but the measure passed without further comment or complaint.

It was probably relevant to this that few MPs (and a minority of voters) were motorcyclists. Commentators acknowledged that motorcyclists might not like compulsion, but many others—such as their families—certainly would.^7^ As well as being a minority, motorcyclists were characterised as particularly daring and mostly very young. This enabled two distinctive arguments in favour of compulsion: that these deaths and injuries were particularly harmful, and that motorcyclists were less than fully competent to make their own decisions. ‘Motor-cycling accidents take the cream of our youth,’ said one member of the House of Lords; another pointed out that ‘these young people, these boys and girls, who rush about our roads’ were exactly those ‘who have everything we want for the future,’ especially ‘[i]f war came again.’^8^ One Conservative MP also argued that a ‘great deal of money is invested in young men, in their education,’ and this was lost when they died prematurely on the roads.^9^ The perceived value to the nation of bold young motorcyclists meant that their life and wellbeing could be situated as something of exceptional importance, worthy of special measures.

Even more significantly for arguments about paternalism, their youth meant that motorcyclists could be presented as less than fully competent to make their own decisions. ‘Many of these youngsters are not sufficiently responsible to recognise the great pain and suffering which they cause to relatives and others,’ Conservative MP Gerald Nabarro had said in 1956. The government therefore had a duty to step in and ‘provide protection for these persons who have not sufficient common sense to protect themselves.’^10^ Arch-libertarian Ronald Bell MP made this even more explicit in the debate over seatbelts some years later, arguing that that the ‘safety helmet legislation – not that I approved of it – overwhelmingly applies to very young people, and a proscription in respect of children has always been accepted by society.’^11^ Here, *all* motorcyclists (who had to be at least 16 years old, or 17 from 1971) became ‘young people’ who became ‘children.’ Along similar lines, Conservative MP Gresham Cooke likened the government’s position in relation to motorcyclists to that of parents towards their children, with one ‘entitled’ to tell the other what to do.^12^ It may be ‘grandmotherly’ to do so, admitted Labour MP Frederick Bellenger, but what was wrong with that? ‘After all, a grandmother knows quite a number of things from long experience.’^13^ Motorcyclists could be positioned as inexperienced and immature: compulsion was therefore simply the right thing to do.

## Paternalism Becomes Contentious

Regulations to penalise motorcyclists for failing to wear helmets then took some years to materialise. It was recognised as unpopular amongst motorcyclists themselves, and road safety efforts focused elsewhere during the 1960s.^14^ The decision to introduce helmet regulations was announced by the Conservative transport secretary John Peyton in February 1973, after 2 years of preparation concerning the standard of helmet to be required.^15^ Some degree of controversy was anticipated: a handful of motoring organisations had indicated their opposition to such ‘an undemocratic interference with the liberty of motor cyclists.’^16^ No press conference was scheduled; a straightforward announcement would ‘stir up enough hornets,’ observed one civil servant, ‘without also exposing the Minister to being openly buzzed at by the captive Press representatives of the motor cyclists.’^17^

In the event, the helmet regulations attracted little press attention, but the muted debate of the 1950s and 1960s flared up in Parliament with surprising vigour. Although the power to make regulations already existed and little could be done to block them, Conservative MP Enoch Powell signalled his vehement disapproval, ‘convinced that a genuinely new and important principle’ of limiting people’s choices purely for their own good was at stake.^18^ He had supporters: as *The Times* reported, protests from ‘[l]egislative purists’ from both main political parties ‘battered the Government mercilessly’ when the Regulations were debated (Noyes 1973). A small but vocal group of about thirteen MPs, including William Hamling of the Labour party along with Ronald Bell and Enoch Powell, frequent allies on the (far) right of the Conservatives, loudly condemned this ‘gross infringement of personal liberty.’^19^

Beyond Parliament, some motorcyclists certainly agreed that this was a highly unwelcome interference in their freedom. Fred Hill accrued numerous fines for failing to wear a helmet which he refused on principle to pay, winning the admiration of Ronald Bell (Bell 1981; *The Times* 1973b). Sikh motorcyclists also protested, petitioned, and refused to pay fines to draw attention to their request for an exemption for those who wore turbans as part of their religious practice—an exemption finally granted three years later (Bebber 2017). Although the government had been aware of the desire for this exemption from the outset, the question of religious objections was not raised by MPs opposing compulsory helmets on the basis of personal freedom.^20^ There is no sign that Bell or Powell, united in their opposition to immigration and the Race Relations Act 1965, gave any support to Sikh activists.

The furore over helmets may have dampened government enthusiasm for a seatbelt law. Perhaps more significantly, the government itself was divided. The Conservative party had come to power in 1970, contrary to most expectations at the time, and by 1973 there was something of a ‘sense of impending apocalypse in Britain’s political class’ (Pemberton 2009, 586). This decade in the UK has often been characterised as one of chaos, crisis and decline. Amidst inflation, recession, and hostile industrial relations, the presence of fragile and minority governments plus increasing political extremism were seen to mark the end of the post-war consensus (Robinson *et al.* 2017). This included profound rifts within the Conservative party itself. As one MP had observed back in 1962, the debate over helmets ‘illustrates a fissure in the Conservative Party. There is the liberalistic Whiggish side, the freedom of the individual and so on, and the paternalistic, benevolent authoritarian on the other side.’^21^ This fissure was very much evident in 1973: Peyton, the transport secretary, was persuaded of the need for a seatbelt law, but his counterpart in the Home Office, Reginald Maudling, was resolutely opposed.^22^ The ‘liberalistic’ side of the party was given particularly loud voice by Ronald Bell, Enoch Powell and others who railed against helmet regulations and were increasingly prepared to oppose their own party.

All this meant that the first attempt to pass a seatbelt law, initiated in 1973 in the House of Lords, generated a profoundly noncommittal reaction from the government. It was interrupted by a sudden general election in February 1974, and when it was reintroduced under the new minority Labour government, those opposed to compulsory seatbelts had begun to marshal their troops. Rising controversy meant that the provision was removed from the Road Traffic Bill, and the parliamentary session ended before its reintroduction could be fully debated.^23^ Between 1974 and 1979, numerous unsuccessful attempts under Labour secured its place as a highly controversial, morally loaded step.

As well as party political instability, this heightened sense of controversy reflects the fact that a seatbelt law ran counter to broader socio-political trends. The 1960s had seen various steps towards reconfiguring the proper role of the state in relation to individual behaviour, with issues like abortion, homosexuality and attempted suicide increasingly viewed as a matter of private rather than public morality. This was always only partial, with the decriminalisation of cannabis, for example, debated on similar grounds but rejected (Seddon 2020). Nevertheless, the language of individual freedom was very much in the air, in political manifestos and everyday conversation as well as calls for drug law reform. Those who opposed a seatbelt law tapped into growing tendencies towards popular individualism, in which many people were ‘increasingly insistent… about defining and claiming their individual rights, identities and perspectives,’ expressing ‘desires for greater personal autonomy and self-determination’ (Robinson *et al.* 2017, 302). Paternalistic legislation could be presented as a retrograde step, founded on the outdated ‘concept that the gentlemen in Whitehall... know best.’^24^

A seatbelt law also ran counter to new trends within public health, in which health was becoming the responsibility of rational, autonomous individuals who should weigh up expert advice and behave accordingly (Clark 2019; Mold *et al.*, 2019). This kind of individual responsibility, with potential to reduce the cost of the struggling National Health Service (NHS), rested on health promotion and choice not compulsion: the ‘fabled gentleman in Whitehall’ may have useful information to share, but should not have the final say.^25^ Similar trends were present in relation to occupational health legislation, which shifted in the 1970s to place responsibility for safety on employers and workers, relying on self-regulation, voluntarism and persuasion (Sirrs 2016). Primary legislation for motorcycle helmets predated the full force of these developments; the prospects for a seatbelt law in the 1970s were much more challenging.

Undoubtedly, the degree of controversy also had something to do with the number of people that would be affected. A last-minute attempt to reintroduce a seatbelt clause in July 1974 met with the complaint that this ‘will, I suppose, affect half the population,’ and therefore demanded a full debate not a snap decision.^26^ Commentators frequently referred to their own and their family’s experiences of collisions and use of seatbelts, signalling their personal investment in the issue (as well as the rise of the anecdote within political rhetoric, as a way of claiming authentic connection to ‘ordinary people’ (Robinson *et al.*, 2017)). Parliamentary votes usually showed that about 60 per cent were in favour of a seatbelt law, but once the idea had become controversial, this was not enough. As one astute commentator observed in 1977, it could only succeed with government support as part of a larger bill. As a stand-alone measure, it was doomed to face the full range of parliamentary obstacles and delaying tactics.^27^

## Refuting Accusations of Paternalism

One such delaying tactic was extremely lengthy parliamentary debate, in which the arguments for and against were repeated time and again. Alongside various subsidiary arguments that waxed and waned over the years to do with safety, comfort, design and enforcement, there were two points that consistently prompted the most energetic disagreement: was this a paternalistic measure that limited a person’s choice for their own good? And if it were, was it nonetheless justifiable? The primary objection was very simply that it was paternalistic and therefore unacceptable. Lord Monson, a crossbench peer and president of the anti-statist Society for Individual Freedom (SiF), made this case clearly and consistently throughout the 1970s: ‘I believe it introduces an additional undesirable element of paternalism, or perhaps one should say “maternalism”, into the law.’ This ran counter to the philosophies of both conservative and liberal traditions alike, in his view, both of which held that ‘an individual should be free to make his or her own mistakes, if indeed mistakes they be, so long as nobody else is harmed in the process.’^28^ Others who were similarly convinced that it was unacceptable ‘big brotherism,’ ‘nannying,’ ‘nursery legislation,’ ‘totalitarian,’ or ‘Fascist/Communist’ included correspondents in the medical and mainstream press as well as an assortment of MPs and peers, mostly (but not all) Conservative.^29^

Although the issue did not elicit any responses from civil liberties organisations or campaigners, these accusations still demanded a reply. Many of those in favour of a seatbelt law argued that it was not such a clear-cut case of paternalism after all. This drew on three points: firstly, that a seatbelt law did not affect a person’s freedom to chose; secondly, that ‘individual freedom’ was perhaps not being removed after all; and thirdly (and most commonly), that the wearing of seatbelts was not something that only affected the wearer, but had a significant impact on others too.

In relation to the question of choice, some argued that freedom of choice would remain: anyone was free to choose to walk instead of using a car.^30^ The president of the Royal College of Surgeons presented the choice somewhat differently in 1978, observing that ‘anyone would be at liberty to ignore or circumvent such laws if he so wished’ (Murley *et al.*, 1978). In an effort to legitimise this element of choice, Labour transport secretary William Rodgers proposed an ‘opt out’ clause for those who objected on principle to being forced to wear a seatbelt—but the idea of being able to opt out of obeying the law was not well received by colleagues concerned with criminal justice.^31^ These discussions had little impact: the choice to break the law was not one that law-makers could realistically acknowledge, nor was the choice *not* to travel by car seen as a meaningful one.

In relation to the removal of ‘individual freedom,’ some argued that drivers and passengers were *not* freely deciding whether to wear a seatbelt in the first place. Echoing academic distinctions between ‘weak/soft’ and ‘strong/hard’ paternalism (Childress *et al.*, 2002; Feinberg 1971), in which ‘weak/soft’ paternalism intervenes in decisions that are not entirely voluntary, such arguments cited the vicissitudes of the human mind and the complexity of social life: passengers did not want to offend drivers by donning a belt, and drivers did not want to worry their passengers; the vast majority were misinformed about risks and benefits, and absolutely everyone was liable to forget. All these circumstances would, it was argued, impede a person’s ability to choose freely. Conservative MP Toby Jessel, who advocated energetically in favour of a seatbelt law following the death of his young daughter in a car collision, held that people were either lazy or mistaken, with ‘incomplete knowledge’ about the dangers on the roads.^32^ Labour MP Jack Ashley agreed that most people were sure that accidents only happened to others and therefore misjudged the true value of a seatbelt.^33^ Decisions were ill-informed, when they were consciously made at all, and so there was no true ‘freedom’ being exercised. This argument, much like any endorsement of the choice to disobey the law, was not taken up very often or pursued very far.

Others tried to distinguish between different kinds of freedom. ‘“Freedom” is a much hackneyed expression,’ observed Labour peer Lord Wells-Pestell. ‘We have to bear in mind that there can be no freedom at all in any society unless there are laws, unless there are restrictions’ which inevitably limit what individuals can do.^34^ Conservative peer Lord Mowbray argued that measures like seatbelt laws enabled safer driving with fewer deaths and injuries, meaning that a more important freedom (to move around by car) was protected.^35^ Jessel argued several times that freedom was ‘not one, indivisible concept.’ He sought to distinguish between ‘great freedoms of speech, conscience and religion’ on one hand, and freedoms that we ‘may not want’ on the other’—such as the freedom to ignore a seatbelt and to be gravely injured or killed as a result. The latter were minor freedoms, in his view, ‘worth sacrificing for health and safety.’^36^

Building on these interrogations of ‘freedom’ was a view expressed only from the Labour benches, that the protection of life and health was necessary for the individual freedom that was being defended so passionately. In essence, this countered calls for negative rights (to be free from government interference) with an argument for positive rights (to live under conditions that enable life and health). MP Bruce George argued that ‘the creation of the Welfare State, public health legislation, health and safety at work legislation and transport legislation’ may restrict freedom in one sense, but created ‘an environment within which I may enjoy another element of freedom.’^37^ Jack Ashley put it bluntly, ‘The basic freedom is life.’^38^ William Molloy spoke more vehemently, drawing on his experience of poverty—or his ‘personal freedom to be out of work’ and ‘nearly starved’—to criticise the valorisation of freedom from state interference. ‘Many people in the valleys of Wales [in the 1930s] were destitute,’ he recalled, ‘but they were in a free land. I have been a little sickened by the argument about personal freedom.’^39^ Freedom here was associated with broader programmes of welfare and social support, but any extension of such programmes—even if only conceptually—was a hard sell in the context of the 1970s, as the welfare state foundered and underwent significant change (Lowe 1994).

By far the most frequent and consistent argument against accusations of paternalism was that wearing a seatbelt did not only affect the wearer, but also the wider community. A 1973 article in the *Lancet* pointed out the potential impact of seatbelt-wearing on ‘the hospitals to which the beltless victims are taken’ and, in emotive terms, on children witnessing unbelted parents maimed or killed. ‘Can one really say that on those occasions no-one else is involved?’ (McKie 1973). The pain and suffering of the families of those injured or killed was certainly not overlooked, but the price paid by emergency responders and medical professionals was a particular talking point. ‘What worries me,’ said David Stoddart in the Commons, ‘is the gory job that nurses, doctors, firemen and policemen have when clearing up after an accident.’^40^ The work of medical and emergency personnel was frequently described in vivid terms, emphasising their ‘considerable anguish and pain’ as well as the cost of their time and expertise.^41^

The costs to others of failing to wear a seatbelt became a regular refrain. There was surely ‘an obligation on each of us to reduce unnecessary calls on the limited resources’ of emergency and medical services, proposed Conservative peer Lord Montagu of Beaulieu, which might include accepting legislation.^42^ The ‘burden’ on the ‘already grossly over-strained’ NHS was oft-cited—unsurprisingly, given the severe financial pressures and reorganisations to which it was being subjected during the 1970s.^43^ Countless correspondents in newspapers and journals agreed that road injuries were not simply ‘the affair of nobody but the victim: the family and the health services cannot be left out of the account’ (Clarke 1977; Personal Liberty versus Common Sense 1980; Seat-Belt Legislation’ 1979; *The Times* 1976; Weston and Paynton 1977). Others highlighted the knock-on effect for scheduled medical procedures, when clinicians had to respond to emergencies.^44^ ‘Dying on the roads is not a private matter,’ the voiceover of an influential television documentary affirmed. ‘It ties up huge resources not just in rescue teams, but of the health services and the police, the courts and the coroners, the repairers and insurers, and the social services’ (The Biggest Epidemic of All Time 1981).

The idea of social cost was extended in numerous directions.^45^ It included the idea that an unbelted driver was more likely to lose control of their car and cause additional collisions;^46^ that the body of an unbelted passenger might harm others within the car (Stewart *et al.*, 1976); the loss of ‘productive work and skill’ when serious injuries and deaths prevented people from ‘mak[ing] a full contribution to society,’ and the cost of providing social care and financial aid to the injured and their dependants.^47^ With the emergence of health economics as a specialist field and the arrival of economic advisers within a wider range of government areas, including transport, some of these social costs could be counted. Labour peer Lord Davies of Leek produced in 1973 a dramatic figure of £115 million for the annual cost ‘to the taxpayer’ of responding to injuries caused by ‘people not wearing a belt;’^48^ the following year his colleague Lord Wells-Pestell made a much more modest claim, that ‘there is ample evidence that the loss of about £40 million a year, including lost production by those killed and in health service costs, is experienced by the community’ as a result of deaths and injuries that might have been prevented by seatbelts.^49^ By 1976, the figure regularly cited had risen to £60 million, rising again to £100 million in 1979–1980.^50^

This focus on the impact of road collisions upon others often shaded into framing people with disabilities, particularly men, as an emotional burden on their families and a financial burden on their communities. ‘What about the wife who might have to suffer for the rest of her life in looking after a husband who is incapacitated, mentally or physically?’ asked MP George Strauss. ‘What about the children who can no longer be dependent on their father?’^51^ This was echoed by Labour MP George Foulkes, with reference to a wife providing lifelong care to a ‘crippled husband,’ and Lord Somers in the House of Lords, who at least included husbands as potential carers of their wives as well.^52^ Labour MP Neil Carmichael put it in ‘brutal’ terms, as he acknowledged: he [sic] who failed to wear a belt ‘may be a burden on the rest of us for the remainder of his life.’^53^

These efforts to foreground the community cost of not wearing a seatbelt met with different kinds of resistance and concern. An editorial in the *Lancet* wondered about the ‘danger’ of emphasising the community costs of unwise personal decisions: ‘it is not such a giant step before communities... start wondering if they any longer want to foot the bill for avoidable mishaps’ (Primary Prevention: The next Stage 1973). What might this mean for the future of public health? This point was picked up in Parliament. Conservative MP Nicholas Ridley objected that presenting a seatbelt law as a way of saving NHS costs was ‘a repulsive argument,’ since it breached the fundamental agreement about shared costs and benefits that underpinned the NHS.^54^ Some did propose that a better solution would be for the unbelted to pay extra towards health and social services;^55^insurance companies, with the support of the courts, had already begun to reduce compensation payments when those injured had not been wearing a belt (*The Times* 1973a).

More broadly, opponents of a seatbelt law protested that seatbelts could not be meaningfully distinguished from any other choice that people made: every single choice had some kind of indirect impact on others. If causing harm to oneself by not wearing a seatbelt should be criminalised, Enoch Powell argued, then ‘there is no aspect of life, inside or outside the home, where punishable offences could not be created on the grounds that they reduced the risk of self-injury.’^56^ References to the awful or absurd prospect of government bans on mountaineering, pot-holing, cigarettes, alcohol or over-eating were frequent.

## Accepting Paternalism

A more concise, but no less regular, perspective was that a seatbelt law certainly *was* paternalistic but nonetheless acceptable. ‘I take the point that the country does not like to be nannied too much,’ said one Conservative peer, ‘but there are points where you ought to nanny people for their own good.’^57^ ‘When people are inadequate,’ Labour MP Ronald Atkins said, ‘they need to be protected against themselves.’^58^ Some framed this as a duty or responsibility, adopting the language of a duty to protect freedom that was used by their opponents. ‘It is our firm duty to carry this Bill,’ insisted the Bishop of London in the House of Lords; it is ‘our collective duty to humanity,’ agreed Labour MP George Robertson, ‘to make sure that the Bill succeeds.’^59^ Racing car driving Jackie Stewart, an enthusiastic campaigner in favour of a seatbelt law, similarly held that it was ‘our social responsibility’ to ensure that as few people as possible were harmed on the roads (Stewart *et al.*, 1976).

The most common line of argument, familiar today, was that it was a small loss of freedom in exchange for a large gain. In this analysis, life and health were more important than the freedom at stake. An editorial in *Lancet* took this position in 1977, dryly commenting that a seatbelt was ‘a mild restraint on individual liberty but a singularly effective restraint on violent forward motion’ (Seat Belts and J.S. Mill 1977). Editorials and correspondents elsewhere agreed that the ‘savings in life, limb and money are so huge and the tiny reduction in personal liberty is so small that really it must make sense’ (Avery 1978; Crawley 1976; *The Times* 1976). Many from all parties in the Lords and Commons agreed.^60^ For the most dedicated opponents of a seatbelt law, the key point was just the opposite: the principle of protecting individual freedom was always more important.^61^

This absolutist defence of individual freedom certainly had its adherents beyond Whitehall, but the freedom not to wear a seatbelt failed to resonate with most civil liberties organisations. By the mid-1970s, there was significant support for a seatbelt law. Medical, safety and motoring organisations were consistently almost unanimously in favour, and surveys indicated that between 60 and 75 per cent of the public were prepared to accept it too (Durie 1976; Willard 1976). As legislative attempt followed legislative attempt, media coverage began to depict mandatory seatbelts as inevitable and to criticise government prevarication (Benson 1979; *Daily Mirror* 1976; McLoughlin 1978; Seat Belts: The Overwhelming Evidence 1977). As early as 1974, *Punch* magazine mocked those who opposed it as old-fashioned and unrealistic. ‘People do not seem to appreciate that it is an Englishman’s inalienable right to get in his own motor car, skid on his own bald tyres, fly through his own windscreen, and leave his own remains spread all over his own bonnet,’ an editorial quipped, and ‘the quicker we realise this, the quicker we shall get India back.’^62^ ‘Do you really regard the right to go through a windscreen as the last bastion of freedom?’ asked a booklet produced by the Royal Society for the Prevention of Accidents.^63^ By 1981, those who still said yes to this question finally lost the battle.

## Conclusions

Compulsory seatbelt wearing came into effect in 1983. Ironically, the transport secretary at the time, Norman Fowler, was a staunch opponent of such a paternalistic measure and investigated every possible alternative, from audio reminders to belt up, to investment in airbags.^64^ However, his tenure saw the third consecutive parliamentary vote in favour of a seatbelt law in less than 2 years, and he faced pressure from the Prime Minister’s office to (be seen to) take action on road safety and to respond to the ‘major and continuing burden on the health and social services (including social security) at a time when we are trying to contain expenditure.’^65^ A seatbelt law had the great advantage of requiring virtually no outlay. Although it remained sufficiently morally contentious to be the subject of a free vote, a majority in both houses voted in favour. As part of a major Transport Bill, its safe passage onto the statute books was secured.

Historical attention to the debates surrounding helmet and seatbelt laws shows that these quintessential examples of paternalism in public health were recognised as such when first proposed, and that this became a problem in the 1970s. Hostility towards policies perceived as paternalistic is variable, affected not only by whose welfare and freedom is involved, but also the broader socio-political context. That which provoked little concern in relation to young motorcyclists in the 1960s became much more controversial a decade later, when it might affect a significant majority of voters. Such controversy was fuelled by political rifts and upheaval, as well as the seatbelt law’s uneasy fit within broader social trends. Opposition to the seatbelt law reflected and responded to the popular individualism of the decade, in which individual choice and freedom was increasingly celebrated.

Supporters of a seatbelt law adopted many elements of current debate around paternalism and public health, albeit often in simple (and sometimes internally inconsistent) terms. They argued that it was *not* paternalistic because an element of choice remained; because greater freedoms were protected; and because the decision not to wear a belt could have significant impacts on others, particularly through the costs to health and social services. Although some expressed concern that this idea could disturb the principles underpinning the NHS, it nonetheless seemed to be one of the most persuasive arguments, and remains a common refrain. In 2015, to mention just one example of recent debate over paternalism and public health, parliamentary debate concerning a tax on sugary drinks included numerous references to the cost to the NHS of the nation’s poor diet.^66^

Supporters of a seatbelt law also argued that it *was* paternalistic but was nonetheless acceptable, because the government *should* tell people what to do at times, and because it would lead to large health benefits in exchange for a small loss of freedom. This final point achieved widespread acceptance, in Parliament and elsewhere, and remains the usual justification for seatbelt mandates (Giubilini and Savulescu 2019; Nuffield Council on Bioethics 2007). The argument that governments have a duty or responsibility to deliver policies that reduce disease, disability and premature death, even if they are paternalistic, is also still made (Gostin and Gostin 2009)—albeit perhaps with less vigour in the context of parliamentary debate.^67^

Although the debate varied little over the 1970s, one anonymous civil servant was probably correct when they wondered whether ‘this is the kind of issue that benefits from continued public discussion, however unpropitious the circumstances.’^68^ The 1970s may have been unpropitious, but such discussion gradually made the prospect of a UK seatbelt law more familiar and its supporters more vocal, while seatbelt laws came into effect around Europe and much of the English-speaking world. By the early 1980s, this constant debate as well as the presence of seatbelt laws elsewhere meant that a sense of inevitability prevailed. Hostility towards the paternalism of a seatbelt law was more the product of the era than the precise nature of the intervention; had primary legislation to make motorcycle helmets compulsory been proposed in the 1970s instead of the 1960s, it seems likely that it would have faced a similarly turbulent time.

## Footnotes

1 Discussion paper from November 1965, in The National Archives, London (hereafter TNA), MT 98/563; Royal Society for the Prevention of Accidents National Road Safety Committee meeting minutes 14 December 1972, in TNA MT 92/556.

2 Consultation responses are in TNA MT 98/778.

3 Hansard HL Deb 28 January 1953 vol 180 col 38. See also Hansard HC Deb 27 October 1952 vol 505 cols 155-6, WA.

4 Hansard HC Deb 31 May 1956 vol 553 col 491.

5 Accident data available in TNA MT 92/206.

6 Hansard HC Deb 17 July 1962 vol 663 col 265.

7 Hansard HC Deb 17 July 1962 vol 663 col 257, 262.

8 Hansard HL Deb 28 January 1953 vol 180 col 34; col 31. See also Hansard HC Deb 31 May 1956 vol 553 col 491, col 493-4; Hansard HC Deb 17 July 1962 vol 663 col 258, cols 263-4, col 280.

9 Hansard HC Deb 17 July 1962 vol 663 col 275.

10 Hansard HC Deb 31 May 1956 vol 553 col 494.

11 Hansard HC Deb 20 July 1979 vol 970 col 2264.

12 Hansard HC Deb 17 July 1962 vol 663 col 275.

13 Hansard HC Deb 17 July 1962 vol 663 col 276.

14 TNA MT 92/257.

15 TNA MT 98/788; MT 92/521; MT 92/522; MT 92/588.

16 Response from National Association of Cycle and Motor Cycle Traders, September 1971, in TNA MT 92/522.

17 Memorandum dated 30 November 1971, in TNA MT 92/522.

18 Letter to John Peyton dated 17 April 1972, in TNA MT 92/588.

19 Hansard HC Deb 5 April 1973 vol 854 col 745.

20 Notes from April 1973 in TNA MT 92/588.

21 Hansard HC Deb 17 July 1962 vol 663 col 275.

22 Memorandum dated 25 May 1971, in TNA HO 310/241.

23 Brief for Legislation Committee dated 28 March 1977, in TNA MT 92/633.

24 Hansard HL Deb 11 June 1974 vol 352 col 369.

25 Hansard HL Deb 26 April 1977 vol 382 col 468.

26 Hansard HC Deb 23 July 1974 vol 877 col 2114.

27 Brief for Legislation Committee dated 28 March 1977, in TNA MT 92/633.

28 Hansard HL Deb 17 December 1973 vol 348 col 106. See also HL Deb 15 December 1980 vol 415 col 946; HL Deb 26 April 1977 vol 382 cols 468-470; HL Deb 11 June 1974 vol 352 cols 366–369; HC Deb 1 March 1976 vol 906 col 949.

29 Hansard HL Deb 17 December 1973, vol 348 cols 106-7; HL Deb 11 June 1974 vol 352 col. 366, 369; HL Deb 25 June 1974 vol 352 col 1344; HC Deb 1 March 1976 vol 906 col 993; HC Deb 20 July 1979 vol 970 col 2212 and 2215; HL Deb 15 December 1980 vol 415 col 943, 947; see also newspaper clippings in TNA MT 102/375.

30 Hansard HL Deb 17 December 1973 vol 348 col 108; HC Deb 28 July 1981 vol 9 col 1039.

31 TNA CAB 129/204/10 and CAB 129/204/12; memorandum dated 25 October 1978 in TNA PREM 16/2282.

32 Hansard HC Deb 1 March 1976 vol 906 col 957.

33 Hansard HC Deb 1 March 1976 vol 906 col 947-8.

34 Hansard HC Deb 11 June 1974 vol 352 col 375-6

35 Hansard HL Deb 28 April 1977 vol 382 col 435.

36 Hansard HC Deb 1 March 1976 vol 906 col 957; also HC Deb 22 March 1979 vol 964 col 1762.

37 Hansard HC Deb 20 July 1979 vol 970 col 2247; see also HC Deb 30 January 1974 vol 868 col 515.

38 Hansard HC Deb 1 March 1976 vol 906 col 946-7; see also HC Deb 22 March 1979 vol 964 col 1731.

39 Hansard HC Deb 1 March 1976 vol 906 col 1006-7; see also HC Deb 20 July 1979 vol 970 col 2237; HL Deb 26 April 1977 vol 382 col 450.

40 Hansard HC Deb 30 January 1974 vol 868 col 515.

41 Hansard HC Deb 1 March 1976 vol 906 col 988. See also cols 955 and 1028 in the same debate.

42 Hansard HL Deb 15 November 1973 vol 346 col 862.

43 Hansard HC Deb 1 March 1976 vol 976; see also cols 906 and 961; HC Deb 20 July 1979 vol 970 col 2237, 2252, 2284; HL Deb 15 December 1980 vol 415 cols 912-3, 926.

44 For example, Roger Stott in Hansard HC Deb 28 July 1981 vol 9 col 1054.

45 See, for example, Hansard HC Deb 21 November 1974 vol 881 col 1645 and HL Deb 17 December 1973 vol 348 col 109-110.

46 Hansard HL Deb 11 June 1981 vol 421 col 339. See also HC Deb 1 March 1976 vol 906 col 931; HL Deb 24 May 1977 vol 383 col 1209 and col 1218; HC Deb 22 March 1979 vol 964 col 1759; HC Deb 20 July 1979 vol 970 col 2217.

47 Hansard HL Deb 15 December 1980 vol 415 col 507. See also HC Deb 22 March 1979 vol 964 col 1741; HL Deb 15 December 1980 vol 415 col 913; HC Deb 1 March 1976 vol 906 col 1024.

48 Hansard HL Deb 17 December 1973 vol 348 col 102.

49 Hansard HL Deb 11 June 1974 vol 352 col 377.

50 Hansard HC Deb 1 March 1976 vol 906 col 928; reiterated in HC Deb 22 March 1979 vol 964 col 1791; HC Deb 20 July 1979 vol 970 col 2218; HL Deb 15 December 1980 vol 415 col 913.

51 Hansard HL Deb 11 June 1981 vol 421 col 343. See also HL Deb 25 June 1974 vol 352 col 1358.

52 Hansard HC Deb 20 July 1979 vol 970 col 2218; HL Deb 25 June 1974 vol 352 col 1358.

53 Hansard HC Deb 20 July 1979 vol 970 col 2204.

54 Hansard HC Deb 25 June 1976 vol 913 col 2094.

55 Hansard HL Deb 17 December 1973 vol 348 col 107.

56 Letter to John Peyton dated 17 April 1972, in TNA MT 92/588.

57 Hansard HL Deb 17 December 1973 vol 348 col 117

58 Hansard HC Deb 22 March 1979 vol 964 col 1766. See also HC Deb 30 January 1974 vol 868 col 515; HL Deb 11 June 1974 vol 352 col 378.

59 Hansard HL Deb 15 December 1980 vol 415 col 918; HC Deb 22 March 1979 vol 964 col 1743.

60 Hansard HL Deb 11 June 1981 vol 421 col 347; HL Deb 26 April 1977 vol 382 cols 456, 488, 512; HC Deb 1 March 1976 vol 906 col 962; HC Deb 22 March 1979 vol 964 col 1731-2.

61 See also, for example, Hansard HC Deb 20 July 1979 vol 970 col 2261; HC Deb 28 July 1981 vol 9 col 1056.

62 Punch magazine, 27 November 1974, clipping in TNA MT 92/578/1.

63 RoSPA leaflet entitled ‘Seatbelt Sense’, p.5, copy in TNA MT 191/131.

64 Meeting note dated 22 June 1979, in TNA MT 191/131.

65 Memorandum from Sir Kenneth Berrill dated 1 June 1979, in TNA PREM 19/3576; Letter from 10 Downing Street to the Ministry of Transport dated 3 June 1979, in TNA PREM 19/3576.

66 Hansard HC Deb 30 November 2015 vol 603 cols 1WH-48WH; Hansard HC Deb 18 April 2017 vol 624 cols 572, 598, 602.

67 Hansard HC Deb 21 December 2010 vol 520 col 1344; HL Deb 20 November 2013 vol 749 cols 408-9, for example.

68 Memorandum dated 3 May 1977 in TNA MT 92/634.
